# Polysialic acid promotes remyelination in cerebellar slice cultures by Siglec-E-dependent modulation of microglia polarization

**DOI:** 10.3389/fncel.2023.1207540

**Published:** 2023-07-10

**Authors:** Lara-Jasmin Schröder, Hauke Thiesler, Lina Gretenkort, Thiemo Malte Möllenkamp, Martin Stangel, Viktoria Gudi, Herbert Hildebrandt

**Affiliations:** ^1^Clinic for Neurology, Hannover Medical School, Hannover, Germany; ^2^Center for Systems Neuroscience Hannover, Hannover, Germany; ^3^Institute of Clinical Biochemistry, Hannover Medical School, Hannover, Germany; ^4^Translational Medicine, Novartis Institute for Biomedical Research, Novartis, Basel, Switzerland

**Keywords:** multiple sclerosis, organotypic cerebellar slice culture, remyelination, polysialic acid (polySia), Siglec-E, microglia, neuroinflammation, immunomodulation

## Abstract

Multiple sclerosis is an inflammatory demyelinating disease of the central nervous system. Spontaneous restoration of myelin after demyelination occurs, but its efficiency declines during disease progression. Efficient myelin repair requires fine-tuning inflammatory responses by brain-resident microglia and infiltrating macrophages. Accordingly, promising therapeutic strategies aim at controlling inflammation to promote remyelination. Polysialic acid (polySia) is a polymeric glycan with variable chain lengths, presented as a posttranslational modification on select protein carriers. PolySia emerges as a negative regulator of inflammatory microglia and macrophage activation and has been detected on oligodendrocyte precursors and reactive astrocytes in multiple sclerosis lesions. As shown recently, polySia-modified proteins can also be released by activated microglia, and the intrinsically released protein-bound and exogenously applied free polySia were equally able to attenuate proinflammatory microglia activation via the inhibitory immune receptor Siglec-E. In this study, we explore polySia as a candidate substance for promoting myelin regeneration by immunomodulation. Lysophosphatidylcholine-induced demyelination of organotypic cerebellar slice cultures was used as an experimental model to analyze the impact of polySia with different degrees of polymerization (DP) on remyelination and inflammation. In lysophosphatidylcholine-treated cerebellar slice cultures, polySia-positive cells were abundant during demyelination but largely reduced during remyelination. Based on the determination of DP24 as the minimal polySia chain length required for the inhibition of inflammatory BV2 microglia activation, pools with short and long polySia chains (DP8–14 and DP24–30) were generated and applied to slice cultures during remyelination. Unlike DP8–14, treatment with DP24–30 significantly improved remyelination, increased arginase-1-positive microglia ratios, and reduced the production of nitric oxide in wildtype, but not in Siglec-E-deficient slice cultures. *In vitro* differentiation of oligodendrocytes was not affected by DP24–30. Collectively, these results suggest a beneficial effect of exogenously applied polySia DP24–30 on remyelination by Siglec-E-dependent microglia regulation.

## Introduction

Multiple sclerosis (MS) is an inflammatory disease of the central nervous system (CNS) that leads to the demyelination of the white matter and axonal damage (Haines et al., 2011; Lassmann, 2018; Correale et al., 2019). Demyelinating lesions also increase in the white matter of the cerebellar cortex, and more than 35% of the total cerebellar cortical area can be affected in progressive MS forms (Kutzelnigg et al., 2005, 2007; Gilmore et al., 2009), causing acute and chronic symptoms such as tremor, ataxia, and dysarthria (Wilkins, 2017). Spontaneous remyelination in MS lesions occurs, mediated by oligodendrocytes arising from the differentiation of oligodendrocyte progenitor cells (OPCs), but this is highly limited and incomplete (Franklin and Simons, 2022). Successful strategies for complete remyelination, in which denuded axons are restored by isolating compact myelin, are currently not available. For the prevention and possibly even reversion of motor dysfunctions and total MS-associated disability, improving remyelination is a promising therapeutic avenue (Franklin and Simons, 2022).

In recent years, numerous studies indicate that besides OPC-autonomous mechanisms of remyelination, microglia, and astrocytes mediate immunomodulation and tissue repair (Franklin and Simons, 2022). In particular, activated microglia together with infiltrating monocyte-derived macrophages engage in proinflammatory crosstalk and phagocytosis of myelin debris during demyelination, while during remyelination, anti-inflammatory polarization of microglia might contribute to tissue repair and immune balance (Mahmood and Miron, 2022).

PolySia is the α-2,8-linked homopolymer of the acidic sugar 5-N-acetylneuraminic acid, the most common sialic acid found in the brain (Schauer, 2009). PolySia chains of variable lengths occur as posttranslational glycan modification of only a few protein carriers, most prominently the neural cell adhesion molecule NCAM (Rutishauser, 2008; Schnaar et al., 2014; Thiesler et al., 2022). PolySia-NCAM is a major regulator of cell surface interactions during brain development and can be presented on both neurons and glial cells, such as OPCs and some types of astrocytes (Rutishauser, 2008; Schnaar et al., 2014; Thiesler et al., 2022).

Notably, transient expression of polySia-NCAM has been detected on reactive astrocytes and OPCs in lysophosphatidylcholine (LPC)-induced lesions of the mouse spinal cord (Nait Oumesmar et al., 1995) and in active human MS lesions (Nait-Oumesmar et al., 2007). Furthermore, polySia is present in demyelinated axons in MS lesions but not in remyelinated axons in shadow plaques, which may prevent successful remyelination (Charles et al., 2002). A dual role of polySia for myelin repair has been derived from the analyses of remyelination after cuprizone-induced demyelination. In the cuprizone model, loss of either of the two polySia synthesizing enzymes, ST8SIA2 or ST8SIA4, compromises (ST8SIA2) or improves (ST8SIA4) remyelination (Koutsoudaki et al., 2010; Werneburg et al., 2017). These seemingly contradictory outcomes could be explained by the sequential expression of the two polysialyltransferases and cell-autonomous effects of polySia-NCAM, promoting early stages but inhibiting late stages of OPC differentiation (Werneburg et al., 2017).

In contrast to the presentation of polySia-NCAM at the cell surface, microglia and macrophages can accumulate an intracellular, Golgi-confined pool of polySia on two other carrier proteins, neuropilin-2 (NRP2) and E-selectin ligand-1 (ESL-1) (Werneburg et al., 2016). As shown *in vitro*, this pool of polysialylated proteins can be released in response to inflammatory activation and dampens the inflammatory response in a negative feedback loop mediated by the inhibitory immune receptor sialic acid-binding Ig-like lectin-E (Siglec-E) (Werneburg et al., 2015, 2016; Thiesler et al., 2021). These data suggest a role of the polySia-Siglec-E axis in balancing microglial activation. Importantly, inhibition of inflammatory microglia activation can also be achieved by the administration of protein-free, soluble polySia with an average chain length (degree of polymerization, DP) of ~50, but not by oligosialic acid with a DP of 3 (Thiesler et al., 2021). Similarly, polySia with an average DP of 20 (avDP20) could inhibit inflammatory microglia and macrophage activation via the human polySia receptor Siglec-11 and was successfully applied to ameliorate laser-induced retina damage and lipopolysaccharide-induced neurodegeneration in transgenic mice expressing Siglec-11 in mononuclear phagocytes (Shahraz et al., 2015; Karlstetter et al., 2017; Liao et al., 2021). However, besides its Siglec-dependent inhibition of microglia and macrophages, polySia avDP20 seems capable of directly interacting with properdin, a positive regulator of the alternative complement pathway, to reduce properdin-mediated complement deposition (Liao et al., 2021).

In the context of myelin repair, the effects of protein-free, soluble polySia have not yet been studied. In this study, we used cerebellar organotypic slice cultures (OSCs), a widely exploited model system to screen compounds for myelin repair (Sekizar and Williams, 2019), with the aim to elucidate the potential of exogenously administered polySia to improve remyelination.

## Materials and methods

### Animals

Animals were bred in the central animal facility of Hannover Medical School (MHH) and underwent routine cage maintenance. Microbiological monitoring was performed according to the Federation of European Laboratory Animal Science Associations (FELASA) recommendations (Guillen, 2012). All procedures were approved by the Lower Saxony State Office for Consumer Protection and Food Safety (LAVES; permission no. 19/3277) and were performed according to international guidelines on the use of laboratory animals.

For pilot experiments, 8–10-week-old C57BL/6J breeding couples were purchased from Charles River Laboratories (Sulzfeld, Germany), and their offspring were killed at postnatal days 9 to 11 (P9–11) to generate OSCs. For primary OPC cultures, Sprague-Dawley rats, purchased from Charles River, were bred at MHH, and dissected brains of P0–P3 neonates were provided by A. Guba-Quint (Cardiology, MHH). Siglec-E knockout mice (B6;129S5-*Siglece*^*tm*1*Lex*^/Mmucd, MMRC), obtained from the Mutant Mouse Resource & Research Center (MMRRC, UC Davis, CA), were backcrossed with C57BL/6 mice for at least 10 generations (Andes et al., 2021). Homozygous *Siglece*^−/−^ and *Siglece*^+/+^ matings were derived from common heterozygous founders.

Genotyping was performed using the following primers: 5′-CTTCCCTATGCCCTAGAACCAT-3′ (DNA287-23) and 5′-CCCTAGGAATGCTCGTCCCGA-3′ (GT-IRES), leading to a 548 bp fragment indicative for the *Siglece* knockout allele, and 5′-GGCCAGTCACTGCGTCTCAT-3′ (DNA287-19) and 5′-CAGTAAGGTTTTGGATAGCCAG-3′ (DNA287-29), resulting in a 414 bp fragment indicative for the *Siglece* wildtype allele.

### Organotypic slice cultures

OSCs from the murine cerebellum were prepared as previously described (Stoppini et al., 1991). Briefly, 350 μm thick parasagittal brain slices from postnatal 9 to 11-day-old (P9–11) C57BL/6J, *Siglece*^−/−^, or *Siglece*^+/+^ mice were cut using a vibratome (Leica VT1000 S Vibrating blade microtome). The slices were cultured on Millicell-CM culture well inserts in 6-well plates (Millipore, Darmstadt, Germany) with a medium containing 50% minimum essential medium (Invitrogen, Carlsbad, USA), 25% Hank's balanced salt solution (Lonza, Verviers, Belgium), 25% horse serum (Invitrogen, Carlsbad, CA), 1% penicillin/streptomycin, 6.5 mg/ml of glucose (Invitrogen, Carlsbad, USA), and 2 mM L-glutamine (Thermo Fisher Scientific, Waltham, USA) for a maximum of 14 days at 37°C and 5% CO_2_. Per animal, eight OSCs were prepared and evenly assigned to the four different treatment groups (see *Results* section). Per cell culture well, four OSCs from two different animals with matched *Siglece* genotypes were co-cultured on one cell culture insert.

**Figure 1:**
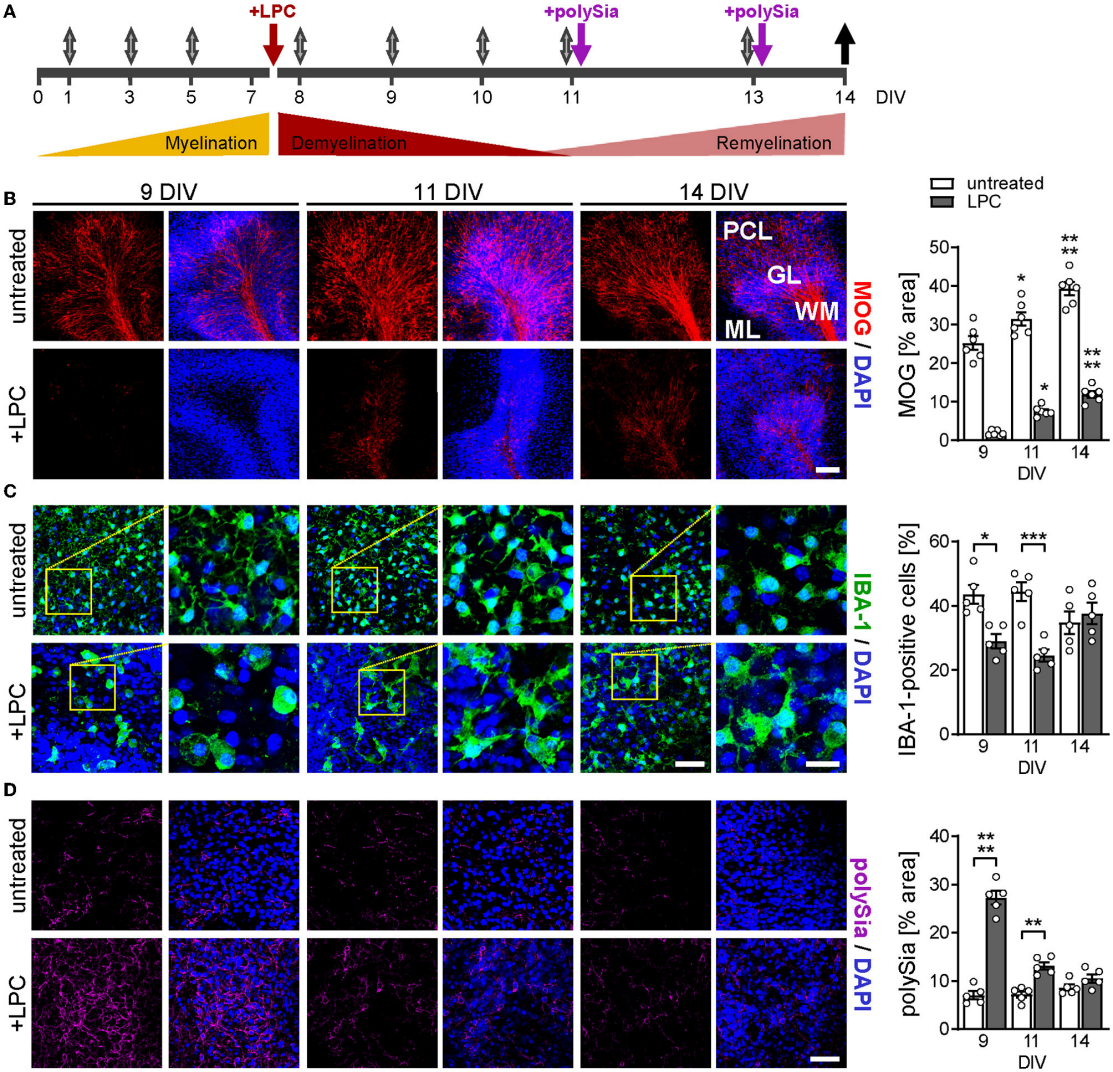
Experimental design and overview of myelin, microglia, and polySia status during de-and remyelination. **(A)** Timeline. Cerebellar slices from P9–P11 mice were cultured for 7 days to complete developmental myelination, and were treated with LPC (7 DIV, red arrow) for white matter demyelination with severe pathology at 9–11 DIV. For polySia treatments (purple arrows), DP8–14 or DP24–30 were added at 11 and 13 DIV to achieve concentrations of 50 and 30μg/ml per slice culture well, respectively. Gray arrows, media exchange, black arrow, sampling of culture media. **(B–D)** Representative micrographs and evaluation of staining for MOG, a marker of terminally differentiated myelinating oligodendrocytes **(B)**, IBA-1 staining for microglia **(C)**, and expression of polySia **(D)** at 9, 11, and 14 DIV, as indicated. Nuclear counterstain with DAPI (blue). Scale bars, 100 μm in **(B)**, 50 μm in **(C)**, overviews, and **(D)**, 20 μm in **(C)**, higher magnification views. Images of MOG display white matter (WM), granular layer (GL), Purkinje cell layer (PCL), and molecular layer (ML). Images of IBA-1 and polySia staining were taken from WM and GL. Morphometric evaluations of MOG **(B)** and polySia **(D)** show individual values and means ± SEM of MOG- or polySia-positive areas in OSCs derived from *n* = 6 or *n* = 5 animals per group, respectively. IBA-1 positive cell numbers **(C)** were evaluated relative to overall cell numbers determined by nuclear DAPI stain, and data represent individual values and means ± SEM of OSCs derived from *n* = 5 animals per group. In **(B–D)**, the two-way ANOVA revealed significant differences, and Tukey's *post-hoc* tests were applied. Significant differences against DIV 9 of the same treatment **(B)** or between untreated and LPC-treated groups of the same DIV **(C**, **D)** are indicated (*P < 0.05; **P < 0.01; ***P < 0.001; ****P < 0.0001).

The slices were cultured for 7 days to allow full developmental myelination. To elaborate on the effect of exogenously added soluble polySia on remyelination, cell culture wells containing cerebellar slices were demyelinated by incubation with LPC (0.5 mg/ml) for 15–17 h after 7 days *in vitro* (DIV). PolySia fractions of defined DP (see below) were applied as detailed in the *Results* section. At 14 DIV, supernatants were collected and OSCs were fixed as described below (for timeline, see Figure 1).

### OPC cultures

OPCs were harvested from mixed glial cultures derived from eight P0–P4 Sprague-Dawley rats per experiment, as described previously (Gingele et al., 2019), and seeded onto poly-L-lysine-coated glass coverslips in a 24-well plate (10^5^ cells per coverslip). OPCs were incubated for 4 h in oligodendrocyte medium (KnockOut^TM^ DMEM/F-12, supplemented with 2 % StemPro^®^ Neural Supplement, and 1% GlutaMAX™ Supplement; all Gibco, Karlsruhe, Germany) with an additional 20 ng/ml of EGF, 20 ng/ml of FGF-1, and 10 ng/ml of PDGF-AA (all Thermo Fisher Scientific) for proliferation (Gingele et al., 2019). Following washing with PBS, OPCs were incubated for 48 h in 300 μl of oligodendrocyte medium devoid of growth factors, supplemented without or with 5 μg/ml of polySia as specified in the *Results* section.

Per OPC preparation, a purity of >95% was determined by double-staining of one differentiated, but otherwise, untreated culture for PDGFR-α (recombinant rabbit mAb, Abcam, Cambridge, UK, 1:200) to detect cells of the oligodendrocyte lineage and for GFAP (mouse monoclonal IgG1, Millipore, Burlington, MA, USA, 1:200) to detect astrocytes (< 2%). In addition, one untreated culture was stained for PDGFR-α together with a CD11b-specific antibody (mouse IgG2a, clone OX-42, Bio-Rad, Hercules, CA, USA, 1:200) to demonstrate < 3% of contamination with microglia. To control cell proliferation, one well of each treatment group was labeled with Alexa Fluor 488-labeled mouse anti–Ki-67 mAb (mouse IgG1, BD Pharmingen, Heidelberg, Germany, 1:100). A rate of 97% Ki-67-positive cells was uniformly observed for all OPC preparations and treatment groups.

### BV2 cell culture

The murine microglial cell line BV2 was cultured as described previously (Thiesler et al., 2021). For inflammatory activation, 5 × 10^4^ cells were seeded in 96-well plates, treated with an activation mix consisting of lipopolysaccharide (LPS, Sigma Aldrich, St. Louis, MO, USA, #L3129, 10 ng/ml), LPS-binding protein (Icell, #C370, 50 ng/ml), and zymosan (InvivoGen, San Diego, CA, USA, #tlrl-zyn, 10 μg/ml) in 200 μl of serum-free medium, and cultured for 24 h in the presence or absence of polySia as specified with the respective results.

### Generation of polySia fractions

PolySia fractions of defined DP were generated by the limited hydrolysis of colominic acid sodium salt (Sigma Aldrich, #C5762) in 100 mM acetic acid. Subsequently, pH was adjusted to 10.5 with NaOH, followed by 48 h incubation at 4°C. Fractions with single DPs were separated as described (Thiesler et al., 2021), and fractions of interest were pooled, desalted by size exclusion applying a 2 kDa cutoff for the isolation of fractions with DP8-14 or a 3 kDa cutoff for the isolation of fractions with DP>14, and followed by lyophilization and storage at −20°C. The DPs of the fractions were assigned using α2,8-linked tetrasialic acid (DP4) as a standard (Nacalai Tesque, Kyoto, Japan, #00642-42).

### Whole-mount immunohistochemistry

OSCs were fixed for 2 h in 4% paraformaldehyde (PFA), followed by washing with PBS and permeabilization for 2 h in PBS with 0.6 % Triton X-100 (Serva, Heidelberg, Germany). After blocking with PBS-containing 0.3 % Triton X-100 and 5 % normal goat serum (NGS) for at least 4 h, the slices were incubated with primary antibodies diluted in PBS with 0.3% Triton X-100 for 48 h by 4°C. After repeated PBS washing, slices were incubated with secondary antibody in PBS/0.3 % Triton X-100 overnight at 4°C. Slices were washed three times with PBS and immediately mounted in VECTASHIELD with 4,6-diamidino-2-phenylindole (DAPI, Vector Laboratories, Burlingame, CA, USA).

The following primary monoclonal antibodies (mAbs) and polyclonal antibodies (pAbs) were used: myelin oligodendrocyte glycoprotein (MOG)-specific hybridoma supernatant (generated from hybridoma cells provided by Christopher Linington, University of Glasgow, UK, 1:2), ionized calcium-binding adaptor molecule 1 (IBA-1)-specific rabbit pAb (Wako Chemicals, Neuss, Germany, 1.200), arginase-1 (ARG-1)-specific goat pAB (Abcam, Cambridge, UK, 1:200), and polySia-specific mouse mAb 735 (Frosch et al., 1985), produced in-house as described (Werneburg et al., 2015) (2 μg/ml). Secondary antibodies were goat anti-mouse Alexa-550, goat anti-rabbit Alexa-488, and donkey anti-goat Alexa-555 (all from Invitrogen).

### Immunocytochemistry

After treatment, OPC cultures were repeatedly washed with PBS, and living cells were incubated for 30 min at 37°C with a differentiation medium containing 1:2 diluted mouse hybridoma supernatants with equal amounts of mouse anti-A2B5 (IgMμ, clone 105) and mouse anti-galactocerebroside (GALC, IgG3, clone IC-07, both from the European Collection of Authenticated Cell Cultures, ECACC, Salisbury, UK). Cells were fixed for 15 min with 4% PFA, incubated for 45 min with Alexa Fluor 488-conjugated goat anti-mouse IgG3, 1:500, and Alexa Fluor 555-conjugated goat anti-mouse IgMμ, 1:500 (both Thermo Fisher Scientific), and mounted in Mowiol^®^ (Merck Millipore, Darmstadt, Germany) with DAPI (Sigma Aldrich, 1:1000).

### Microscopy, image acquisition, morphometry, and cell counting

Images were acquired on an LSM 900 confocal microscope equipped with an Airyscan 2 detector and Zen 2012 (blue edition) software (Carl Zeiss Microscopy, Göttingen, Germany). For morphometric evaluation of MOG labeling, images of 3–4 individual lobules within the cerebellar cortex of each OSC, and from two OSCs per animal, were evaluated, after background subtraction, by calculating the percentage of immunopositive areas in frames of 350 × 350 μm using ImageJ Software (Fiji, U. S. National Institutes of Health, Bethesda, Maryland). The numbers of IBA-1 and ARG-1 immunopositive cells within the white matter and the internal granular cell layer of a cerebellar lobule were quantified in 3–4 lobules per OSC and from two OSCs per animal, using the bioimaging analysis platform QuPath (Bankhead et al., 2017), in which cells were segmented and subsequently classified. The cell segmentation model was trained under visual control on a set of five images per condition using the deep learning-based StarDist library (Schmidt et al., 2018).

Immunopositive cells in OPC cultures were counted at 20x magnification using an Olympus BX41 fluorescence microscope and cellSens software (Olympus, Tokyo, Japan).

### Nitric oxide determination

To assess nitric oxide (NO) production, nitrite, the stable reaction product of NO, was determined by the colorimetric Griess assay. Supernatants from the culture slices were collected per well, cleaned by precipitation with acetonitrile (80%v/v), and centrifuged at 16.000x*g* (15 min, 4°C). The supernatants were dried at RT. After rehydration in 10% of the original volume, the sample was mixed 1:1 with Griess reagent, prepared in-house as described (Thiesler et al., 2021), and incubated for 20 min (RT). Absorbance at 540 nm was determined against a reference wavelength of 620 nm, nitrite concentrations were calculated from a standard curve (Promega, Wisconsin, USA), and values were normalized to the mean of untreated wildtype controls.

### Statistical analysis

GraphPad Prism version 8.02 was used for statistical analysis (GraphPad, San Diego, USA). The one- or two-way ANOVA followed by Tukey's *post-hoc* tests were applied as indicated. Normality and equality of variances were assessed using the Shapiro–Wilk and the Brown–Forsythe test, respectively. To meet the assumption of normal distribution, the ROUT method with a false discovery rate of Q = 1% was used to eliminate four outliers from the determinations of nitrite in OSC supernatants (Figure 4). Values are presented as arithmetic means ± standard error of the mean (SEM). A *P*-value of < 0.05 was considered statistically significant.

## Results

### PolySia is transiently elevated during LPC-induced demyelination

Figure 1A illustrates the timeline of LPC-induced OSC de- and remyelination and the experimental treatment scheme. At the onset of the culturing period (P9–11), cerebellar myelination is not yet completed. To permit the progression of developmental myelination, the slices were cultured for 7 days before being treated with 0.5 mg/ml of LPC for 15–17 h, leading to the ablation of white matter at 9 DIV, as illustrated by the absence of staining for MOG, a marker of terminally differentiated myelinating oligodendrocytes (Figure 1B). Spontaneous remyelination was detected at 11 DIV and proceeded until 14 DIV, the endpoint of the study (Figure 1B). Staining for microglia revealed initial activation at 9 DIV, indicated by clustering, process retraction, and adoption of an amoeboid shape, followed by an almost complete reversion to a ramified morphology, indicative of homeostatic microglia (Figure 1C). Compared to untreated controls, the numbers of IBA-1-positive microglia were transiently decreased after LPC treatment. This is in good agreement with previous data, showing a similar decline of microglia due to necroptosis early after LPC application to cerebellar OSCs and prior to the onset of remyelination in mice injected with LPC into the corpus callosum (Lloyd et al., 2019).

Consistent with the transient polySia expression by astrocytes and OPCs during LPC-induced demyelination of the spinal cord *in vivo* (Nait Oumesmar et al., 1995), abundant polySia immunoreactivity was detected in cerebellar lobules during the phase of demyelination (9 DIV), which rapidly declined to low levels at 11 and 14 DIV (Figure 1D). Accordingly, during remyelination and treatment with exogenously applied soluble forms of protein-free polySia, only little intrinsic, cellular polySia was present in the OSCs. Thus, the OSC model appears suited to test the effects of exogenous polySia on remyelination.

### PolySia has a critical degree of polymerization to attenuate the inflammatory response of microglia

**Figure 2:**
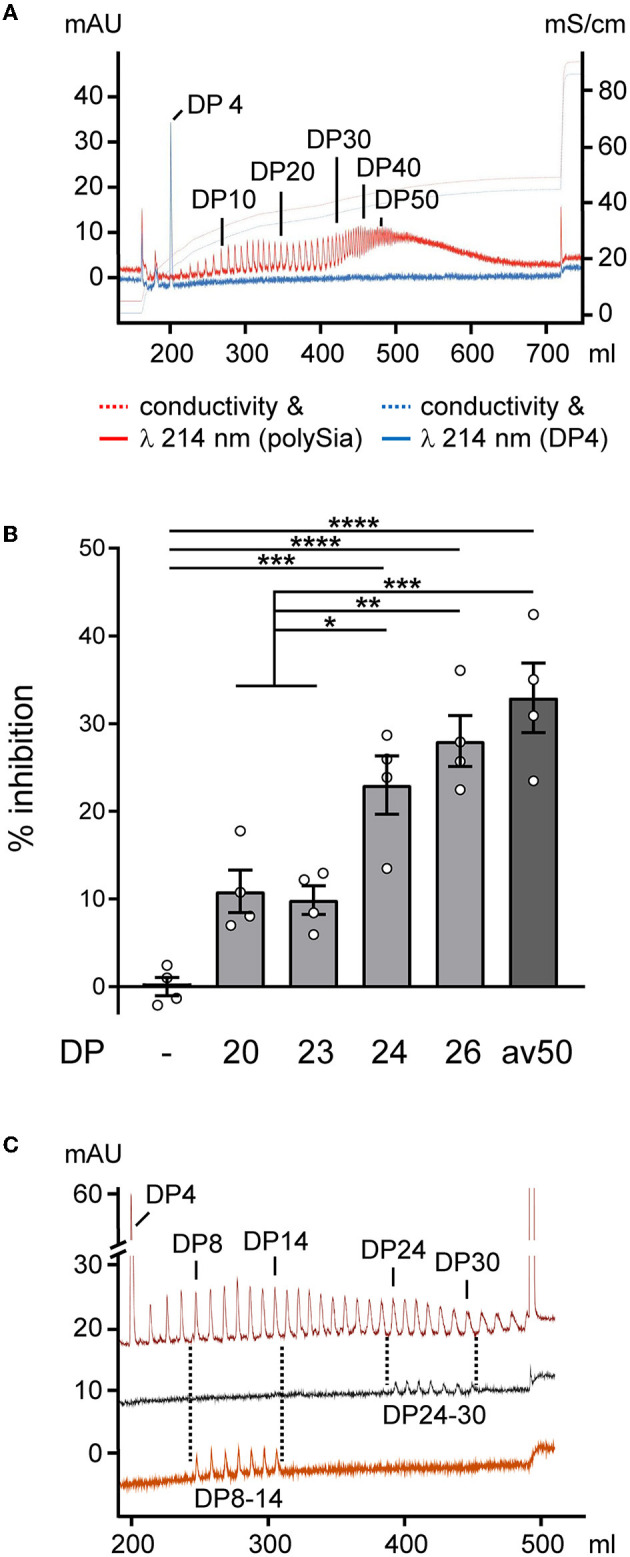
Production of polySia fractions with defined DPs and effects on proinflammatory activation. **(A)** Analytical anion exchange chromatography of the polySia batch used as a starting material. DPs of individual peaks were determined by spiking with α2,8-linked tetrasialic acid (DP4, blue spectrum). *N*-acetyl groups of sialic acid were detected by absorption at 214 nm. **(B)** BV2 microglia were activated by the application of 10 ng/ml of LPS, 50 ng/ml of LPS-binding protein, and 10 μg/ml of zymosan (LLZ; joint activation of TLR4 and TLR2) in serum-free medium and treated without (–) or with polySia of the indicated DP, or with polySia with an average DP of ~50 (avDP50, 250 nM, each). NO production was assessed by the detection of the NO breakdown product nitrite using the colorimetric Griess assay, and % inhibition of LLZ-induced NO production is plotted. The inhibition of ~30% in the presence of FPLC-purified avDP50 corresponds to the inhibition obtained by polySia applied to LPS-induced primary or stem cell-derived murine microglia (Werneburg et al., 2015, 2016), or to LPS-induced BV2 cells (Thiesler et al., 2021). Per group, individual values and means ± SEM from *n* = 4 independently treated cultures are plotted. The one-way ANOVA revealed significant differences (*P* < 0.0001), and Tukey's *post-hoc* tests were applied. Significant group differences are indicated (*P < 0.05; **P < 0.01; ***P < 0.001; ****P < 0.0001). **(C)** Spectra of polySia (with DP4 added) after partial hydrolysis on a preparative scale (upper trace), and of pooled fractions with DP24–30 (middle) and DP8–14 (lower) analyzed after lyophilization, determination of yield by weighing, and rehydration. Salt gradient for elution same as in **a**, but with a step at 432 mM to 1 M NaCl.

Aiming at treatment with polySia of defined chain lengths, polySia fractions consisting of defined DPs were separated (Figure 2A) and used to test for the inhibition of LPS-induced NO production as a wellestablished readout for inflammatory activation and the Siglec-E-dependent inhibitory effect of polySia avDP50 in microglial BV2 cells (Thiesler et al., 2021). As shown in Figure 2B, only polySia fractions with a DP of 24 and more showed efficient inhibition, comparable to the effect of polySia with avDP50. For polySia treatment of OSCs, we used pools consisting of polySia DP24–30, which can inhibit proinflammatory-activated microglia, and DP8–14, which cannot (Figure 2C).

### PolySia DP24–30 improves remyelination

**Figure 3:**
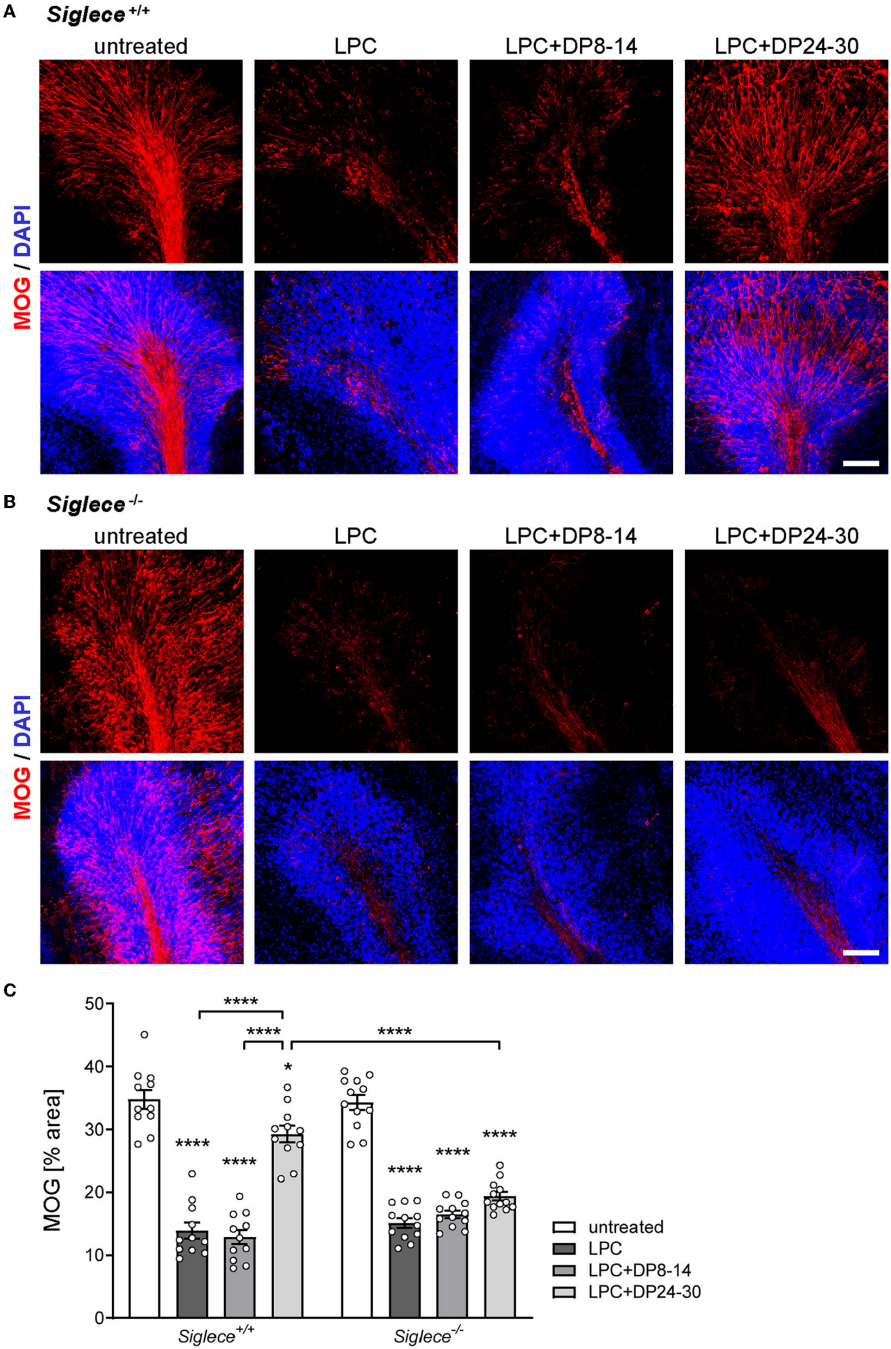
PolySia DP24–30 improves remyelination in a Siglec-E-dependent manner. OSCs from Siglec-E wildtype and knockout mice (*Siglece*^+/+^, *Siglece*^−/−^) were treated as indicated, and fixed at 14 DIV (for details, see Figure 1). **(A, B)** Representative images of MOG staining. Scale bars, 100 μm. **(C)** Morphometric evaluation. Data represent individual values and means ± SEM of MOG-positive areas in OSCs derived from *n* = 11–18 animals per group. Per animal eight OSCs were prepared, and two OSCs were assigned to each of the treatment groups. Per OSC, 3–4 frames of 350x 350 μm, each representing a different cerebellar lobule, were acquired and evaluated by morphometric determination of the MOG-positive area in percent of the total area. The two-way ANOVA revealed significant differences, and Tukey's *post-hoc* tests were applied. Significant differences within each genotype group and for selected comparisons between genotypes are indicated. Asterisks assigned to a single treatment group indicate significant differences against the untreated control of the same genotype (*P < 0.05; ****P < 0.0001).

Effects of exogenously applied polySia fractions with DP8–14 and DP24–30 on remyelination were analyzed in a pilot experiment with OSCs from wildtype C57BL/6J mice. Morphometric evaluation of MOG revealed a significant improvement in remyelination after the application of DP24–30, while DP8–14 had no effect (Supplementary Figure 1). A comparative approach with *Siglece*^+/+^ and *Siglece*^−/−^ OSCs (Figure 3) reproduced this outcome for the *Siglece*^+/+^ groups (Figures 3A, C). In sharp contrast, polySia DP24–30 had no effect on remyelination in *Siglece*^−/−^ OSCs (Figures 3B, C). As evident in the wildtype C57BL/6J and *Siglece*^+/+^ groups (Supplementary Figure 1; Figure 3A), remyelination after the application of DP24–30 was not confined to the white matter but also detected throughout the internal granular and Purkinje cell layers of the cerebellar lobules.

Because the improved remyelination in the presence of polySia DP24–30 was dependent on the presence of the microglia-specific polySia receptor Siglec-E, and because DP8–14, which was not able to inhibit the inflammatory activation of cultured microglia, had no effect, it can be assumed that the impact of polySia DP24–30 is mediated by Siglec-E dependent inhibition of proinflammatory microglia.

### PolySia DP24–30 inhibits NO production and augments the fraction of arginase-1-positive microglia during remyelination

**Figure 4:**
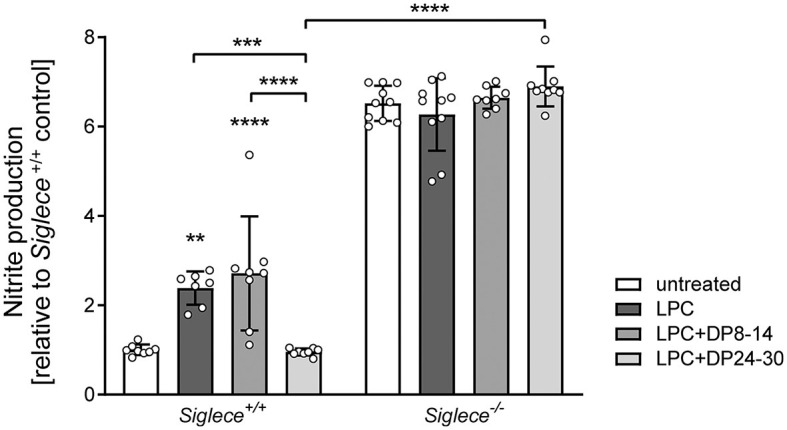
PolySia DP24–30 inhibits NO production of OSCs in a Siglec-E-dependent manner. NO production was determined by nitrite detection (Griess assay) in supernatants of OSCs treated with LPC and polySia DP8–14 or polySia DP24–30 as indicated. Each well contained four OSCs from two different animals, and supernatants were collected at 14DIV. Data represent individual values and means ± SEM of *n* = 8–10 values per group normalized to the level of untreated *Siglece*^+/+^ controls. To meet the assumption of normal distribution, the ROUT method with a false discovery rate of Q = 1% was used to eliminate four outliers (one in *Siglece* ^+/+^, LPC, two in *Siglece* ^−/−^, DP8–14, and one in *Siglece* ^−/−^ DP24–30). The two-way ANOVA revealed significant differences, and Tukey's *post-hoc* tests were applied. Significant differences within each genotype group and for selected comparisons between genotypes are indicated. Asterisks assigned to a single treatment group indicate significant differences against the untreated control of the same genotype (**P < 0.01; ***P < 0.001; ****P < 0.0001).

NO production is a hallmark of proinflammatory microglia activation and is widely accepted as a mediator of oligodendrocyte cytotoxicity and demyelination in the CNS (Smith and Lassmann, 2002; Liñares et al., 2006; Merrill et al., 2009). Therefore, and to corroborate the assumed inhibitory effect of polySia DP24–30 on inflammatory microglia activation during remyelination, NO production was determined by measuring the stable NO breakdown product nitrite in OSC supernatants at 14 DIV. NO production was significantly increased in LPC-treated cerebellar OSCs derived from C57BL/6J and *Siglece*^+/+^ mice and this was completely reverted by treatment with polySia DP24–30, but not with DP8–14 (Supplementary Figure 1 for C57BL/6J, Figure 4 for *Siglece*^+/+^, respectively). In the absence of the inhibitory immune receptor Siglec-E, however, NO production was strongly elevated in all treatment groups and neither affected by LPC nor polySia treatment (Figure 4), consistent with results obtained previously for *Siglece*^−/−^ BV2 microglia (Thiesler et al., 2021).

**Figure 5:**
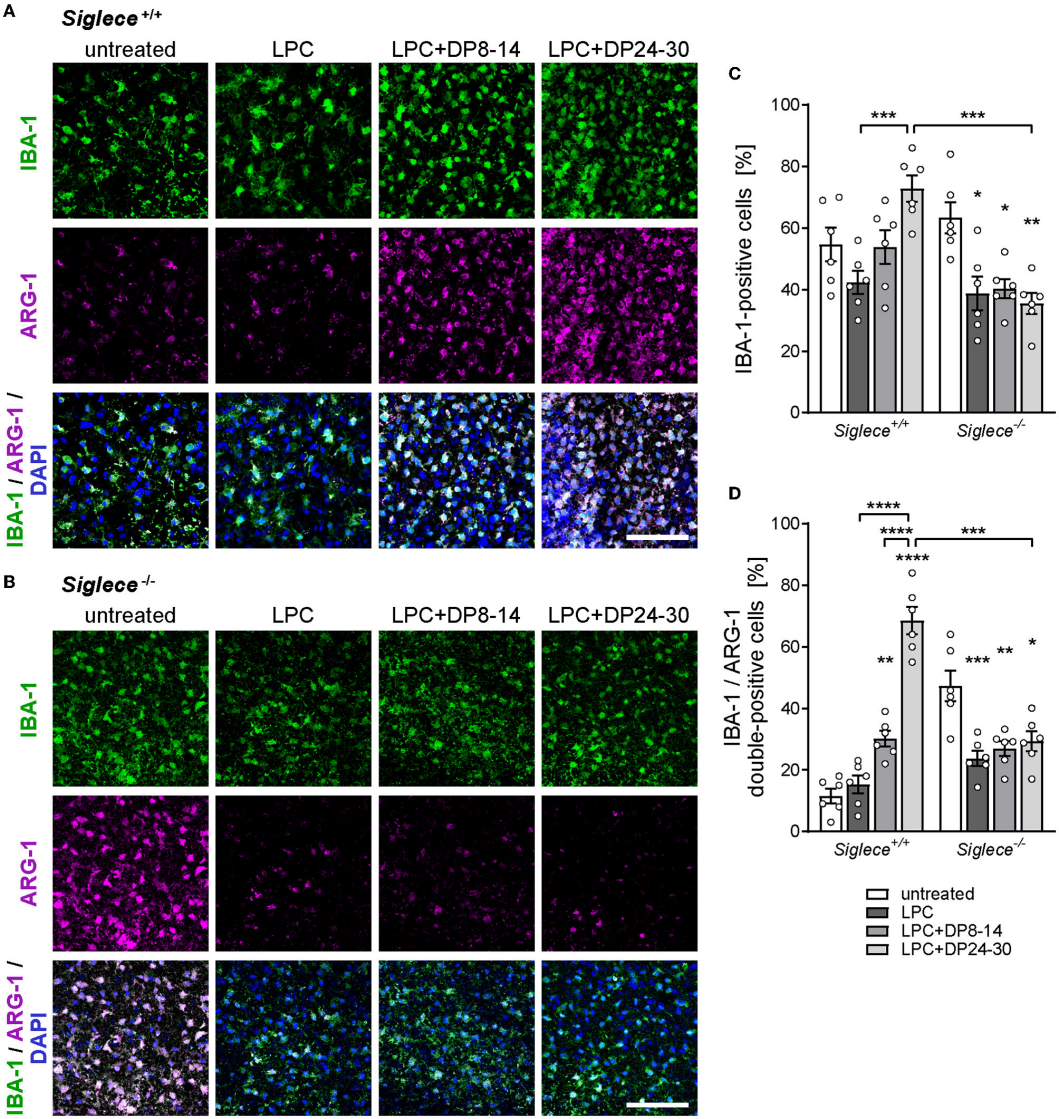
PolySia DP24–30 drives anti-inflammatory microglia polarization in a Siglec-E-dependent manner. **(A, B)** Representative images of double immunostaining for IBA-1 (green) and arginase-1 (ARG-1, magenta) in white matter areas of OSCs from *Siglece* ^+/+^ **(A)** and *Siglece* ^−/−^ mice **(B)**, treated with LPC and polySia DP8–14 or polySia DP24–30 as indicated, and fixed at 14 DIV (for details, see Figure 1). Nuclear counterstain with DAPI (blue). Scale bars, 50μm. **(C, D)** Quantification of IBA-1-positive **(C)** and IBA-1/ARG-1 double-positive cells **(D)** relative to overall cell numbers determined by nuclear DAPI stain. Data represent individual values and means ± SEM of OSCs derived from *n* = 6 animals per group. In **(C, D)**, the two-way ANOVA revealed significant differences, and Tukey's *post-hoc* tests were applied. Significant differences within each genotype group and for selected comparisons between genotypes are indicated. Asterisks assigned to a single treatment group indicate significant differences against the untreated control of the same genotype (*P < 0.05; **P < 0.01; ***P < 0.001; ****P < 0.0001).

We next sought to study the expression of arginase-1 in remyelinating white matter areas. Arginase-1 is an opponent of NO production and a marker of anti-inflammatory, protective microglia, and macrophage phenotypes that are essential for remyelination (Miron et al., 2013). In contrast to slightly enhanced overall numbers of IBA-1-positive microglia, numbers of IBA-1 and arginase-1 double-positive microglia were dramatically increased in LPC-treated OSCs derived from *Siglece*^+/+^ mice and exposed to polySia DP24–30 (Figures 5A, C, D). Again, polySia DP8–14 had no significant impact. Unexpectedly, the numbers of IBA-1 and arginase-1 double-positive cells were significantly higher in untreated *Siglece*^−/−^ as compared to *Siglece*^+/+^ OSCs (Figures 5B, D). This might be linked to the slight, but not significant increase in IBA-1-positive microglia numbers observed in this group (Figure 5C). The simultaneous reduction of the IBA-1-positive and the IBA-1 and arginase-1 double-positive cells in LPC-treated *Siglece*^−/−^ OSCs supports this assumption. Unlike the strong increase of NO production in all *Siglece*^−/−^ OSCs irrespective of treatment (see Figure 4), the reduction in cell numbers after LPC treatment indicates that microglia in *Siglece*^−/−^ OSCs still react to the LPC-induced demyelination. Moreover, an increase in arginase-1-positive cells may counterbalance the increase in NO production by *Siglece*^−/−^ OSCs during LPC-induced de- and remyelination, which may explain the unaltered level of remyelination in otherwise untreated *Siglece*^−/−^ OSCs (see Figures 4B, C). Importantly, however, in contrast to *Siglece*^+/+^ OSCs, numbers of IBA-1- and arginase-1-positive microglia were not increased in remyelinating *Siglece*^−/−^ OSCs treated with polySia DP24–30 (Figures 5B–D). Together, these data indicate that polySia DP24–30 not only inhibits the proinflammatory activation of microglia during remyelination but also induces a Siglec-E-dependent shift in polarization toward a protective phenotype that supports remyelination.

### PolySia DP24–30 has no direct impact on OPC differentiation

**Figure 6:**
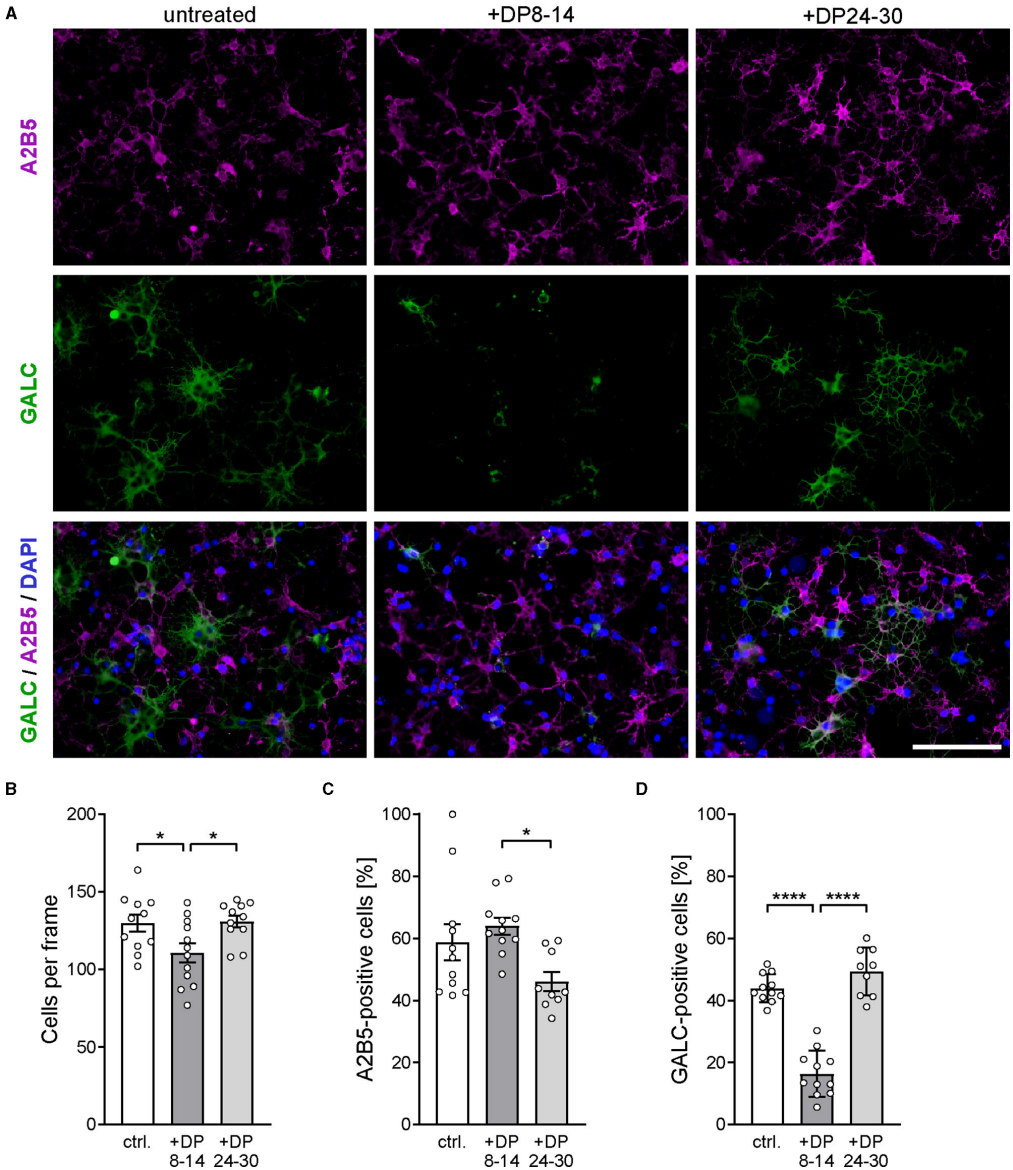
PolySia DP24–30 has no direct impact on OPC differentiation. **(A)** Representative images of primary rat OPC cultures stained for A2B5 (magenta) and GALC (green) after 2 days of *in vitro* differentiation in the presence of polySia DP8–14 or polySia DP24–30, as indicated. Nuclear counterstain with DAPI (blue). Scale bars, 50 μm. **(B–D)** Absolute cell numbers per frame **(B)**, and relative numbers of A2B5 **(C)** or GALC-positive cells **(D)**. Data represent individual values and means ± SEM of *n* = 9–11 independent OPC cultures per condition. OPCs were obtained from overall three OPC pools prepared from eight neonatal rats, each. Per OPC culture well, 15–20 frames (150 × 200 μm) were evaluated. The one-way ANOVA revealed significant differences (*P* < 0.0001 for GALC; *P* = *0.0223* for A2B5), and Tukey's *post hoc* tests were applied. Significant group differences are indicated (*P < 0.05; ****P < 0.0001).

As shown in previous studies, the level of polySia attached to proteins on the surface of OPCs has a direct effect on their differentiation toward mature oligodendrocytes (Werneburg et al., 2017). Therefore, the observed beneficial effect of soluble polySia DP24–30 on remyelination could also be, at least in part, mediated by a cell-autonomous stimulation of OPC differentiation. To address this possibility, we studied the effect of polySia DP24–30 on the differentiation of primary rat OPC cultures. Differentiation was assessed by monitoring the numbers of immature A2B5-expressing OPCs and mature GALC-expressing oligodendrocytes after 48h in the differentiation medium without or with the addition of polySia fractions (Figure 6).

Compared to untreated controls, polySia DP24–30 had no effect on the total number of cells (Figure 6B) and on the numbers of A2B5-positive OPCs or GALC-positive oligodendrocytes (Figures 6C, D). Unexpectedly, DP8–14 had a negative impact on particularly the numbers of GALC-positive cells. This contrasts with the complete absence of polySia DP8–14-mediated effects on remyelination and microglia polarization.

In summary, these results demonstrate that exogenously applied polySia DP24–30 promotes remyelination and provide strong evidence that this effect is mediated by a Siglec-E-dependent shift from a proinflammatory toward an anti-inflammatory microglia phenotype.

## Discussion

PolySia is emerging as a novel regulator of microglia activity, but, so far, its intricate role in developmental myelination and remyelination has been mostly assigned to regulatory functions in OPC differentiation mediated by polySia attached to NCAM at the cell surface (reviewed in Thiesler et al., 2022). A negative feedback regulation of inflammatory microglia activation by the release of polySia through ectodomain shedding of polySia-NRP2 and polySia-ESL-1, and its perception by the inhibitory immune receptor Siglec-E has been demonstrated in studies with cultured microglia (Werneburg et al., 2015, 2016; Thiesler et al., 2021). Evidence that this mechanism may be relevant under pathophysiological conditions *in vivo* was derived from the identification of polysialylated NRP2 and ESL-1 and the immunohistochemical detection of intracellular polySia accumulations in the Golgi compartment of injury-induced microglia in mouse brain OSCs (Werneburg et al., 2016) and from the sequence of polySia appearance and disappearance in microglia activated by a cortical stab wound (Thiesler et al., 2021). Furthermore, the application of soluble, protein-free polySia with avDP20 led to reductions of laser-induced retina damage and lipopolysaccharide-induced neurodegeneration in transgenic mice expressing human Siglec-11 in mononuclear phagocytes (Karlstetter et al., 2017; Liao et al., 2021).

These studies provided proof of principle for the potential of therapeutic polySia applications. In the *in vivo* studies, however, it remained unresolved, which of the competing or complementary polySia interactions with transgenic Siglec-11, endogenous Siglec-E, or, possibly, with properdin reducing complement deposition independent of Siglec-mediated microglia regulation (Shahraz et al., 2022), were decisive for the improved outcome. In contrast, several lines of evidence indicate that the beneficial impact of polySia on remyelination, as demonstrated in the current study, is mediated by the modulation of microglia activity. First, we demonstrate that only polySia with DP24–30 but not with DP8–14 is able to elicit the observed effect. This is consistent with our finding that DP24 is the critical minimal polySia chain length required to inhibit the inflammatory activation of BV2 microglia under controlled conditions *in vitro*. Second, the inability of polySia DP24–30 to improve remyelination in OSCs from *Siglece*^−/−^ mice demonstrates that its effect depends entirely on the presence of Siglec-E, which is a specific immune receptor of cells of the myeloid lineage and therefore confined to microglia as the only resident immune cell population of the brain parenchyma and white matter in OSCs derived from the previously uninjured brain (Zhang et al., 2004; Linnartz-Gerlach et al., 2014; Kierdorf et al., 2019). Third, in remyelinating OSCs, polySia DP24–30 profoundly reduced the production of nitric oxide, a hallmark of proinflammatory microglia activation, and promoted the appearance of arginase-1-positive microglia, indicative of anti-inflammatory polarization, shown to be essential for efficient remyelination (Miron et al., 2013). Fourth, in contrast to polySia presented at the cell surface, polySia DP24–30 had no effect on the differentiation of primary cultured OPCs.

Unexpectedly, polySia DP8–14 had an adverse impact on the *in vitro* differentiation of OPCs without affecting cell numbers. At first sight, this appears consistent with the need to downregulate polySia during OPC differentiation toward myelinating oligodendrocytes, but this has been assigned to an inhibitory effect of polySia on myelin formation by steric and electrostatic cell surface repulsion which is unlikely to be conveyed by soluble, short but not long polySia fragments (Fewou et al., 2007, 2019; Bakhti et al., 2013). Furthermore, polySia DP8–14 had no inhibitory impact on the partial remyelination of cerebellar OSCs within 7 days after LPC-induced demyelination. However, the short, soluble polySia fragments may interfere with the promotion of OPC differentiation by endogenous or forced expression of polySia on the OPC surface (Werneburg et al., 2017). Based on experiments with OPCs lacking the polysialyltransferase ST8SIA2, it has been hypothesized that the presence of polySia on OPCs promotes their transition to oligodendrocytes by modulating ligand binding and activation of receptors such as the platelet-derived growth factor receptor alpha (PDGFRα) (Szewczyk et al., 2017). Downstream signaling of PDGFRα is decisive for OPC proliferation but the receptor is rapidly internalized upon ligand binding, downregulated during oligodendrocyte differentiation, and absent on mature oligodendrocytes (Fekete and Nishiyama, 2022). Possibly, the short soluble polySia fragments interfere with this process, but the observed effect of polySia DP8–14 on OPC differentiation under *in vitro* conditions requires further investigation.

Numerous studies demonstrate that a shift of microglia polarization from a proinflammatory toward an anti-inflammatory phenotype supports remyelination, pinpointing microglia as an attractive therapeutic target (Franklin and Simons, 2022; Mahmood and Miron, 2022). The underlying mechanisms are not entirely clear, but, as shown in cerebellar OSCs, involve microglial secretion of growth factors such as activin-A, which contributes to remyelination by directly activating activin receptor signaling on OPCs (Miron et al., 2013). In contrast, indirect mechanisms via modulation of astrocyte activity appear unlikely (Gingele et al., 2019).

There have been many successes in the treatment of MS, but so far all licensed therapeutics aim to modulate the peripheral immune system (Baecher-Allan et al., 2018; Derfuss et al., 2020). Some experimental approaches have addressed remyelination in MS (Gingele and Stangel, 2020); however, no treatment addresses microglia specifically, although there are suggestions that drugs such as teriflunomide may, in addition to the effect on lymphocytes, modulate oligodendrocytes and microglia (Wostradowski et al., 2016; Göttle et al., 2023). Direct modulation of microglia may be an attractive approach to support endogenous myelin repair by an additional mode of action combined with the immunomodulation of the peripheral immune system.

A limitation of our study is that it focused on a model of remyelination that is not reflecting the autoimmune origin of MS. On the other hand, only this approach allows a clear dissection of the underlying mechanism with respect to the remyelination process. Furthermore, there is a need to close the putative translational gap between the mouse model and the human situation concerning differences in the polySia responsive inhibitory Siglec receptors. Pending questions concern the minimal chain length required for polySia interactions with human Siglec-11 as compared to murine Siglec-E. Furthermore, it remains open, if polySia chains longer than DP14 and shorter than DP24 may have additional Siglec-independent effects on remyelination, such as interference with complement activation by properdin, as suggested for polySia avDP20 (Shahraz et al., 2022). Another limitation concerns the question of the role of Siglec-E during spontaneous remyelination in LPC-treated OSCs. The comparable level of remyelination in otherwise untreated *Siglece*^+/+^ and *Siglece*^−/−^ OSCs observed at 14 DIV could be the result of a balance between positive and negative Siglec-E-mediated effects and it will need more extended, longitudinal comparisons to answer this question.

Taken together, this study provides strong evidence for the crucial role of the polySia-Siglec axis in microglial immune balance with beneficial effects on the remyelination process. Based on the profile of polySia chain lengths that are able to modulate microglia activity and polarization, we suggest the controlled application of polySia DP24–30 as a promising strategy to improve myelin repair.

## Data availability statement

The raw data supporting the conclusions of this article will be made available by the authors, without undue reservation.

## Ethics statement

The animal study was reviewed and approved by Lower Saxony State Office for Consumer Protection and Food safety (LAVES; permission no. 19/3277).

## Author contributions

HT, HH, L-JS, MS, and VG: conceptualization. L-JS, VG, HT, LG, and TM: methodology. L-JS, HT, and LG: data acquisition. L-JS, HT, TM, and HH: data analysis and interpretation. L-JS, HT, and HH: writing—original draft. L-JS, HT, HH, VG, LG, TM, and MS: writing—review. All authors contributed to the article and approved the submitted version.
