# Corrigendum: Polysialic acid promotes remyelination in cerebellar slice cultures by Siglec-E-dependent modulation of microglia polarization

**DOI:** 10.3389/fncel.2023.1275048

**Published:** 2023-08-23

**Authors:** Lara-Jasmin Schröder, Hauke Thiesler, Lina Gretenkort, Thiemo Malte Möllenkamp, Martin Stangel, Viktoria Gudi, Herbert Hildebrandt

**Affiliations:** ^1^Clinic for Neurology, Hannover Medical School, Hannover, Germany; ^2^Center for Systems Neuroscience Hannover, Hannover, Germany; ^3^Institute of Clinical Biochemistry, Hannover Medical School, Hannover, Germany; ^4^Translational Medicine, Novartis Institute for Biomedical Research, Novartis, Basel, Switzerland

**Keywords:** multiple sclerosis, organotypic cerebellar slice culture, remyelination, polysialic acid (polySia), Siglec-E, microglia, neuroinflammation, immunomodulation

In the published article, there was an error in Figure 6 as published. In panel A, the micrographs in columns 2 and 3 were labeled incorrectly. The corrected Figure 6 and its caption appear below.

**Figure 6:**
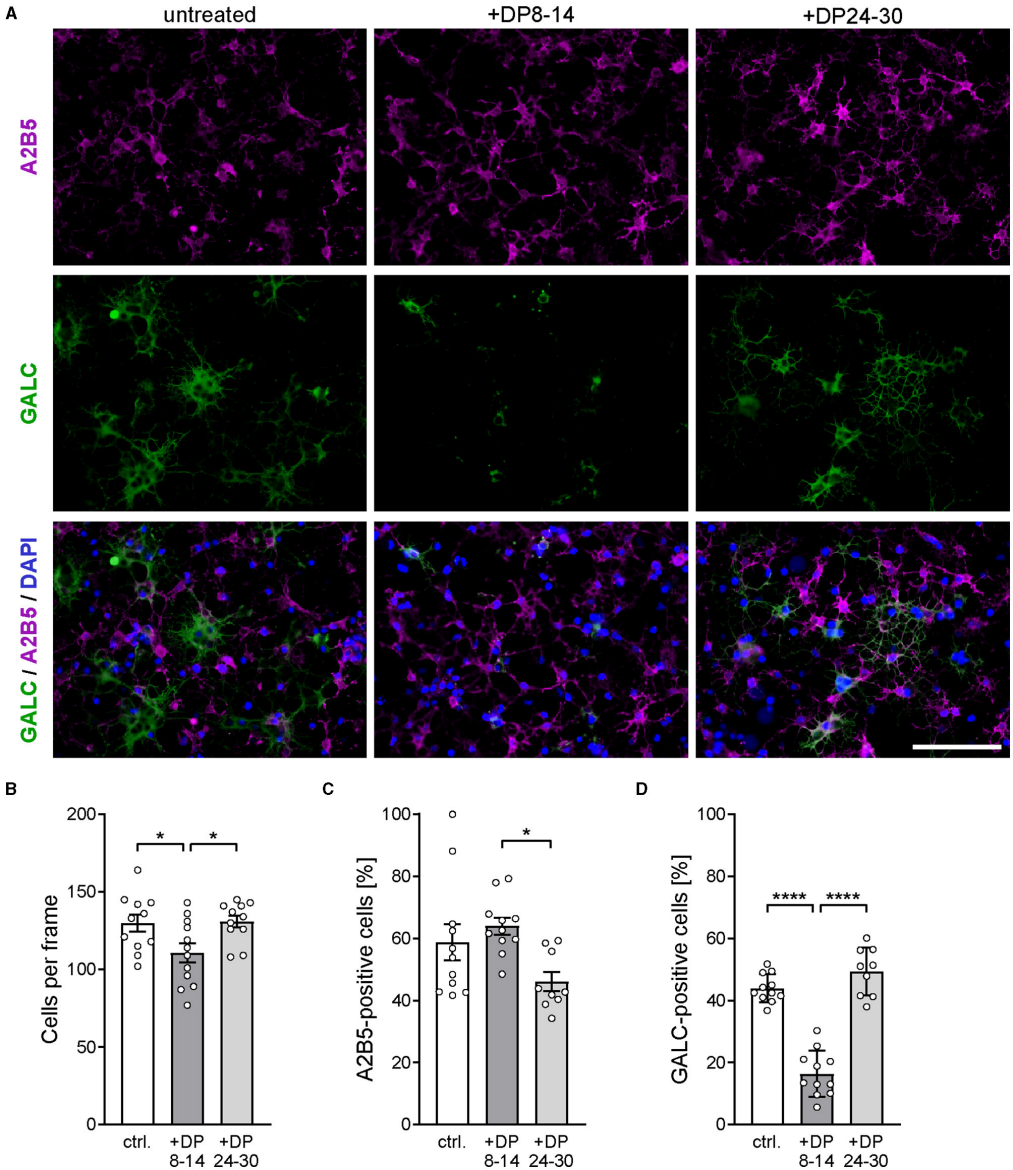
PolySia DP24–30 has no direct impact on OPC differentiation. **(A)** Representative images of primary rat OPC cultures stained for A2B5 (magenta) and GALC (green) after 2 days of *in vitro* differentiation in the presence of polySia DP8–14 or polySia DP24–30, as indicated. Nuclear counterstain with DAPI (blue). Scale bars, 50 μm. **(B–D)** Absolute cell numbers per frame **(B)**, and relative numbers of A2B5 **(C)** or GALC-positive cells **(D)**. Data represent individual values and means ± SEM of *n* = 9–11 independent OPC cultures per condition. OPCs were obtained from overall three OPC pools prepared from eight neonatal rats, each. Per OPC culture well, 15–20 frames (150 × 200 μm) were evaluated. The one-way-ANOVA revealed significant differences (*P* < 0.0001 for GALC; *P* = 0.0223 for A2B5), and Tukey's *post hoc* tests were applied. Significant group differences are indicated (*P < 0.05; ****P < 0.0001).

The authors apologize for this error and state that this does not change the scientific conclusions of the article in any way. The original article has been updated.

