# Supplementary material for: Polysialic acid promotes remyelination in cerebellar slice cultures by Siglec-E-dependent modulation of microglia polarization
Source: Front Cell Neurosci. 2023 Jul 10;17:1207540. doi: 10.3389/fncel.2023.1207540 (PMC10365911; doi:10.3389/fncel.2023.1207540)
Supplement: Supplementary file 1 [file Image_1.PDF]

Supplementary Figure S1

Polysialic acid promotes remyelination in cerebellar slice cultures by Siglec-E-dependent modulation of microglia polarization

Lara-Jasmin Schröder, Hauke Thiesler, Lina Gretenkort, Thiemo Möllenkamp, Martin Stangel, Viktoria Gudi, and Herbert Hildebrandt

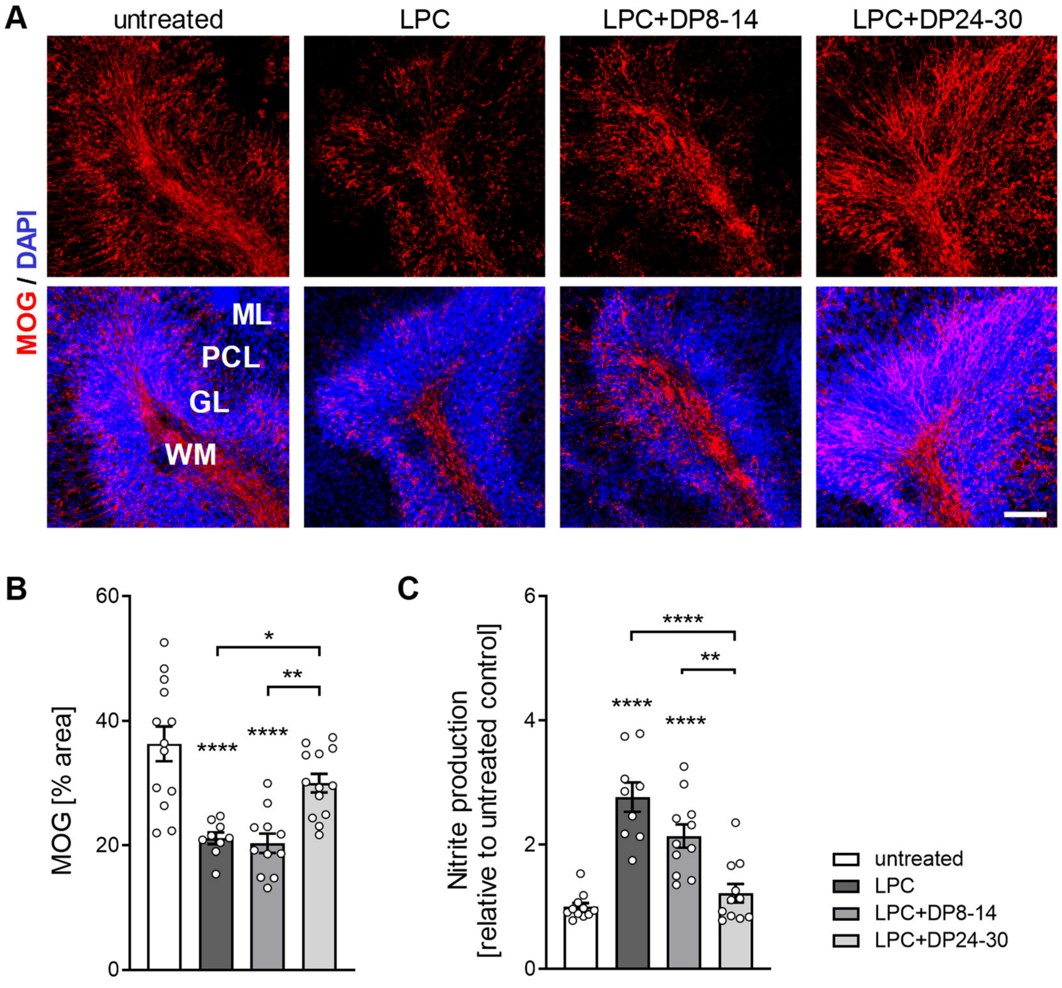

Figure S1. Pilot experiment on the effect of polySia on remyelination and NO production of OSCs derived from C57BL6J mice.

(A) Representative images of MOG staining of cerebellar OSCs at 14 DIV. Scale bar, 100µm. Conditions as indicated. WM, white matter; GL, granular layer; PCL, Purkinje cell layer; ML, molecular layer. **(B)** Morphometric evaluation. Data represent means ± SEM of MOG-positive areas in percent of the total area in frames positioned in the lobules of cerebellar OSCs derived from n=9-13 animals per group (for details see Fig. 3). **(C)** NO production of cerebellar OSCs determined by nitrite detection (Griess assay) in the supernatants (for details, see Fig. 4). Data represent means ± SEM of n=9-11 values per group normalized to the level of untreated controls. In **(B)** and **(C)**, one-way ANOVA revealed significant differences and Tukey's *post hoc* tests were applied. Significant differences between groups are indicated. Asterisks assigned to a single treatment group indicate significant differences against the untreated control (*, $P < 0.05$; **, $P < 0.01$; ****, $P < 0.0001$).
